# Hyperactivity and Motoric Activity in ADHD: Characterization, Assessment, and Intervention

**DOI:** 10.3389/fpsyt.2014.00171

**Published:** 2014-11-28

**Authors:** Caterina Gawrilow, Jan Kühnhausen, Johanna Schmid, Gertraud Stadler

**Affiliations:** ^1^Faculty of Science, Department of Psychology, Eberhard Karls Universität Tübingen, Tübingen, Germany; ^2^German Institute for International Educational Research (DIPF), Frankfurt, Germany; ^3^Center for Research on Individual Development and Adaptive Education of Children at Risk (IDeA), Frankfurt, Germany; ^4^LEAD Graduate School, Eberhard Karls Universität Tübingen, Tübingen, Germany; ^5^Psychology Department, Columbia University, New York, NY, USA

**Keywords:** accelerometers, bifactor models of ADHD, ADHD, hyperactivity–impulsivity, physical activity, support vector machines

## Abstract

The aim of the present literature review is threefold. (1) We will review theories, models, and studies on symptomatic hyperactivity and motoric activity in attention-deficit/hyperactivity disorder (ADHD). (2) Another focus will be on assessment methods that have been proven to be effective in the detection of hyperactivity and motoric activity in children, adolescents, and adults with and without ADHD and emerging areas of research in the field of ADHD. We will compare subjective methods (i.e., rating scales) and objective methods (i.e., accelerometers). (3) Finally, physical activity intervention studies aiming at a modification of activity and overactive behavior will be summarized that seem to be promising candidates for alleviating hyperactivity symptoms in children, adolescents, and adults with ADHD.

## Introduction

Hyperactivity and a general increase in motoric activity with respect to amount/frequency and variability of activity/movements are main symptoms of both the combined and the hyperactive–impulsive type of attention-deficit/hyperactivity disorder (ADHD). Children with ADHD fidget with hands and feet, have difficulties remaining seated, run about, or climb excessively, and have difficulties to engage in activities quietly (1). Children with ADHD show increased physical activity (PA) both during the day and the night (2). Thus, research indicates that in one-third of children with ADHD and with an even higher prevalence in adults with ADHD sleep disorders (i.e., daytime sleepiness, insomnia, delayed sleep phase syndrome, fractured sleep, restless legs syndrome, and sleep disordered breathing) are common (3). Whereas children without ADHD usually outgrow hyperactivity around the age where they enter elementary school, hyperactivity remains present in children who receive an ADHD diagnosis. This leads to severe problems during structured school activities and interactions with parents, teachers, and peers. In adolescents and adults with ADHD, hyperactivity remains as a symptom, leading to, for instance, extreme restlessness and feelings of always being on the go or driven by a motor. Hyperactivity as one of the core symptoms of ADHD is thus leading to maladaptive cognitive and social functioning and disturbed well-being.

Counterintuitively though, children and adolescents with ADHD appear to be less likely to engage in regular vigorous PA and organized sports (4). Research only begins to investigate possible barriers that might constitute underlying reasons for this physical inactivity. One of the reasons might be that due to the ADHD symptoms of inattentiveness and impulsivity, affected children are easily distracted by or respond impulsively to alternative activities [e.g., watching TV (5)]. Another reason might be that deficits in executive functions that potentially underlie the ADHD symptomatology lead to difficulties initiating and maintaining PA (4, 6). Furthermore, it seems that children are at risk for not being physically active when they receive no ADHD treatment [i.e., medication with methylphenidate, MPH (7)].

While inattentiveness as a core symptom has been investigated in numerous studies, no clear characterization of symptomatic hyperactivity and motoric activity in children, adolescents, and adults with ADHD exists. In addition, there are no guidelines for the assessment and intervention of motoric activity in ADHD (8).

Therefore, aims of the present literature review are (1) to review studies on symptomatic hyperactivity and motoric activity in ADHD on different developmental stages. This is important because hyperactivity as a symptom and its possible differentiation from other symptoms as for instance inattentiveness might alter over the lifespan. (2) Another focus will be on subjective and objective assessment methods that have been proven to be effective in the detection of hyperactivity and motoric activity in ADHD. This is important because there is a need for adequate and efficient detection methods of hyperactivity in clinical settings. As we will explain in more detail, clinical diagnoses often rely on retrospective parental and teacher reports on hyperactive behavior shown by children and adolescents. (3) Finally, PA intervention studies aiming at a change or modification of activity and overactive behavior that seem to be promising candidates for alleviating hyperactivity symptoms in ADHD will be summarized.

## Diagnostic Criteria of Symptomatic Hyperactivity and Motoric Activity in ADHD

Hyperactivity constitutes a core symptom of ADHD that represents one of the most common disorders in childhood and adolescence, with approximately 5.3% of school-aged individuals being affected (9). The Diagnostic and Statistical Manual of Mental Disorders (DSM-5) classifies ADHD as a neurodevelopmental disorder and lists 18 symptoms on two dimensions (see Table 1), namely, (1) hyperactivity–impulsivity and (2) inattention that manifest in three possible main presentations: (a) predominantly hyperactive/impulsive presentation (314.01), (b) predominantly inattentive presentation (314.00), and (c) combined presentation (314.01). A diagnosis requires at least six symptoms of hyperactivity–impulsivity and/or inattention in childhood and adolescence, and at least five symptoms in adulthood to be present in two or more settings (e.g., at home, school/work, with friends) for at least six months, and to interfere with developmental level and functioning.

**Table 1:** **Hyperactivity–impulsivity and inattention symptoms according to DSM-5 (1)**.

**Hyperactivity–Impulsivity**
1. Often fidgets with or taps hands or feet or squirms in seat
2. Often leaves seat in situations when remaining seated is expected (e.g., leaves his or her place in the classroom, in the office or other workplace, or in other situations that require remaining in place)
3. Often runs about or climbs in situations where it is inappropriate. (Note: In adolescents or adults, may be limited to feeling restless.)
4. Often unable to play or engage in leisure activities quietly
5. Is often “on the go,” acting as if “driven by a motor” (e.g., is unable to be or uncomfortable being still for extended time, as in restaurants, meetings; may be experienced by others as being restless or difficult to keep up with)
6. Often talks excessively
7. Often blurts out an answer before a question has been completed (e.g., completes people’s sentences; cannot wait for turn in conversation)
8. Often has difficulty waiting his or her turn (e.g., while waiting in line)
9. Often interrupts or intrudes on others (e.g., butts into conversations, games, or activities; may start using other people’s things without asking or receiving permission; for adolescents and adults, may intrude into or take over what others are doing)
**Inattention symptoms**
1. Often fails to give close attention to details or makes careless mistakes in schoolwork, at work, or during other activities (e.g., overlooks or misses details, work is inaccurate)
2. Often has difficulty sustaining attention in tasks or play activities (e.g., has difficulty remaining focused during lectures, conversations, or lengthy reading)
3. Often does not seem to listen when spoken to directly (e.g., mind seems elsewhere, even in the absence of any obvious distraction)
4. Often does not follow through on instructions and fails to finish schoolwork, chores, or duties in the workplace (e.g., starts tasks but quickly loses focus and is easily sidetracked)
5. Often has difficulty organizing tasks and activities (e.g., difficulty managing sequential tasks; difficulty keeping materials and belongings in order; messy, disorganized work; has poor time management; fails to meet deadlines)
6. Often avoids, dislikes, or is reluctant to engage in tasks that require sustained mental effort [e.g., schoolwork or homework (for older adolescents and adults, preparing reports, completing forms, reviewing lengthy papers)]
7. Often loses things necessary for tasks or activities [e.g., school materials, pencils, books, tools (wallets, keys, paperwork, eyeglasses, and mobile telephones)]
8. Is often easily distracted by extraneous stimuli (for older adolescents and adults, may include unrelated thoughts).
9. Is often forgetful in daily activities (e.g., doing chores, running errands; for older adolescents and adults, returning calls, paying bills, keeping appointments)

*Behavioral examples added to symptom descriptions in DSM-5 compared to DSM-IV are given in square brackets. Symptoms 1–6 from the hyperactivity–impulsivity dimension denote symptoms classed as hyperactivity. Symptoms 7–9 from the hyperactivity–impulsivity dimension denote symptoms classed as impulsivity*.

## Current Psychological Theories of Hyperactivity and Motoric Activity in ADHD

Several psychological theories exist that address the question of what gives rise to symptomatic hyperactivity and motoric activity in ADHD. In our review we outline three current theoretical conceptions: (1) the State Regulation Model, (2) Multiple Pathway Theories, and (3) the Dynamic Developmental Theory of ADHD.

The State Regulation Model suggests that clinical levels of ADHD symptomatology can be traced back to a deficit in keeping optimal activation states (10, 11). Building up on the cognitive-energetic theory of Sanders (12) and Sanders and van Duren (13) the model assumes that a given person’s activation level increases in situations where the presentation rate of stimuli is high, and decreases in situations where the presentation rate of stimuli is low. To reach optimal levels of performance and counteract overactivation under high stimulus presentation rates as well as underactivation under low stimulus presentation rates, the allocation of extra effort (i.e., effortful control) is necessary. Several studies at various developmental stages have shown that individuals with ADHD show greatest performance deficits compared to individuals without ADHD not under medium event rates but under fast (14, 15) and slow (15, 16) event rates when the effortful allocation of extra effort would have been necessary for optimal performance. Correspondingly, the State Regulation Model postulates ADHD symptoms, including hyperactivity, to increase or decrease relative to a given person’s respective state that requires effortful control. Thus, in the context of this theoretical model, levels of hyperactivity are not seen as stable across situations but to become increasingly present under low activation states as an attempt of self-stimulation and under high activation states as a behavioral sign of overactivation.

Multiple pathway conceptions (17–19) postulate that there are several distinct developmental influences (i.e., ‘pathways’) that converge onto a core symptom expression of ADHD but with remaining specificities (20). Today, the most prominent multiple pathway conceptions is the Triple-Pathway Model (19) that suggests dissociable *timing*, *inhibition*, and *delay deficits* to give rise to highly heterogeneous expressions of ADHD symptoms. With regard to timing, the model suggests that individuals with ADHD display deficits in, for example, the discrimination of durations and the adequate anticipation of time intervals. Inhibition deficits refer to impaired abilities of individuals with ADHD to inhibit responses in inappropriate situations (21) such as awaiting turn in communications. Delay deficits denote the aversion and enhanced negative emotional reactions to situations characterized by (temporal) delays [e.g., Ref. (22)] such as waiting for further instructions in the classroom. Within this theoretical framework, the symptom of hyperactivity is conceptualized as a behavioral attempt to attenuate negative subjective experiences of delay where delay cannot be circumvented (23).

The Dynamic Developmental Theory of ADHD (24) suggests a hypofunctioning mesolimbic dopamine branch to underlie altered reinforcement of novel behavior and insufficient extinction of behavior that has previously been reinforced. It is assumed that the critical time frame for a reinforcer to take effect is shorter in individuals with ADHD compared to individuals without ADHD. Accordingly, socially desirable behavior is frequently not positively reinforced, and undesirable behavior not negatively reinforced in time. The theory predicts that (gradually developing) hyperactivity stems from a combination of altered positive reinforcement and deficient extinction processes, leading to an increased and accumulating number of behavioral responses that may display in excess motoric activity.

## Structural Accounts of Hyperactivity and Motoric Activity in ADHD

Despite broad consensus about what classifies as hyperactivity in the context of ADHD and several theoretical approaches to its underlying causes, the question of how the symptom relates to impulsivity and inattention has been one of the main areas of scientific debate in this research field for the last 20 years. Respective research questions are (a) is hyperactivity essentially just an expression of one underlying ADHD condition? (b) Is hyperactivity distinguishable from, yet related to impulsivity and/or inattention? (c) Or is a combination of those two perspectives possible? These questions concern our understanding of hyperactivity, how it relates to functional impairment and, by association, what kind of treatment may work and for whom. Important statistical methods to address the ongoing debate are factor analyses, which directly concern the measurement structure underlying ADHD symptoms and thus the question of coherence and distinctness between hyperactivity, impulsivity, and inattention. Factor models that have been discussed are (a) the one-factor model, which assumes one underlying unitary symptom domain, (b) the correlated factor models, which assume distinct symptom domains that are correlated, and (c) bifactor models, which incorporate both an underlying unitary symptom domain and additional specific independent symptom domains.

A one-factor model assumes that there is a single dimension underlying hyperactivity as well as impulsivity and inattention. No structural distinction is made in this model between hyperactivity and other symptom domains. However, there has been abundance of empirical evidence from factor-analytic investigations convincingly showing that this factor model does not represent an adequate conceptualization [e.g., Ref. (25–27)].

Correlated factor models emphasize the separability of hyperactivity from inattention and/or impulsivity (28). They conceptualize separate, yet related, latent constructs with either two factors (i.e., representing hyperactivity–impulsivity and inattention) or three factors (i.e., representing hyperactivity, impulsivity, and inattention) without a common core, which give rise to the phenotypic representation of ADHD.

Factor-analytic studies until the beginning of the 21st century found strong support for correlated factor models being better statistical representations of the symptom structure than the one-factor model [e.g., Ref. (29–34)]. Direct comparisons of the correlated two-factor model separating hyperactivity–impulsivity and inattention into two separate dimensions, and three-factor models separating the dimensions of hyperactivity and impulsivity besides inattention are somewhat inconclusive: Whereas many studies found support for differentiating two latent symptom dimensions (26, 29–31, 35–37), others pointed to the superior fit of models with three latent symptom dimensions that emphasize the separability of hyperactivity and impulsivity [e.g., Ref. (33, 34, 38)]. However, the more parsimonious two-factor model is usually favored because separating hyperactivity from impulsivity tends to improve the overall model fit only slightly and the latent factors of hyperactivity and impulsivity are usually highly correlated (39). Nevertheless, the question has been brought up as to whether the relative underrepresentation of symptoms of impulsivity in DSM (i.e., three) compared to the number of symptoms of hyperactivity (i.e., six; see Table 1) and limited psychometric properties—which limit statistical power to confirm a specific factor of impulsivity—may also explain some of the conflicting results between the studies mentioned above (30, 37, 39). Overall, it has been proposed that support for the validity of the correlated factor models comes from differential associations of the symptom domains with criterion variables of functional impairment (e.g., externalizing and internalizing behaviors). However, subscale sum scores for different symptom domains may not only represent domain specific symptom variation but also variation that can be traced to an underlying general symptom domain. Thus, such differential associations with measures of functional impairment could be compounded with influences from an underlying core symptom domain.

More recently than the one-factor model and correlated factor models, bifactor models have been proposed. They simultaneously account for both the common variance (i.e., coherence) between hyperactivity and the other core symptoms of ADHD with a latent general (g) ADHD factor, and the unique separable variance of hyperactivity, impulsivity, and inattention with specific domain factors (s) that are independent (i.e., orthogonal) from the general ADHD factor. Thus, to a greater extent than correlated factor models, bifactor models promote the common variance between symptom domains suggesting a unitary core construct underlying all ADHD symptoms, while endorsing additional covariation that manifests in orthogonal, specific symptom factors. During the last years, a number of studies have compared the more traditional correlated factor models with bifactor models and generally supported the superior model fit of the latter across a wide range of age groups (i.e., children, adolescents, adults), informants (i.e., self, parent, teacher, clinician ratings), methods of measurement (i.e., rating scales, interviews), and target populations [i.e., clinical and community samples (28, 39–48)]. Nevertheless, substantial inconsistency remains with regard to the question of whether a specific factor of hyperactivity can be distinguished from a specific factor of impulsivity (28, 40, 45, 47, 48). Just as discussed for the correlated factor models, the relative scarcity of impulsivity items may limit the power to detect a separate specific factor representing these symptoms separately from hyperactivity.

In sum, and although further studies and possibly the development of further items to assess impulsivity are needed to shed light on the question of separability between hyperactivity and impulsivity, a bifactor model framework seems to be a valid account of hyperactivity in the context of ADHD and its phenotypic representation for the following reasons: First, studies addressing the question of the development of ADHD across the lifespan reveal that it shows a substantial degree of stability and in many cases persists into adulthood (49), even though the specific symptom manifestation of this disorder may change with development [e.g., Ref. (50)]. This suggests a generic component, which lies at the core of the disorder and is stable over time, along with additional specific manifestations that may fluctuate (43). Due to the lack of a ‘common core’ in correlated factor models, which assume interrelated but conceptually independent symptom domains, they lack explanatory value for such a generic component. Second, quantitative genetic research (i.e., twin and adoption studies) suggest that there are sets of genes that exclusively influence hyperactivity and sets of genes that influence all domains of ADHD symptoms [(51, 52); for a review of quantitative genetic research on ADHD see (53)]. A bifactor model that represents a general ADHD symptom factor as well as independent specific symptom factors may be especially well suited to account for these findings. Third, the bifactor model has been suggested to be in line with current etiological models [e.g., Ref. (28, 43, 54)] such as multiple pathway conceptions (17–19), which postulate that there are several distinct developmental influences (i.e., ‘pathways’) that converge onto a core symptom expression, while specificities do remain (20).

## Symptomatic Hyperactivity and Motoric Activity in ADHD on Different Developmental Stages

Excessive motor activities are a first precursor of ADHD and often observed by parents during toddlerhood, even though they are hardly distinguishable from highly variable normative behaviors during this developmental period. Most frequently, impairing levels of hyperactivity are identified during the primary school years with a majority of children (approximately 60–85%) continuing to meet diagnostic criteria of ADHD throughout childhood and into adolescence and adulthood [e.g., Ref. (55)]. Notably, predominantly boys show symptomatic hyperactivity [e.g., Ref. (56)]. Sparse research addressing the trajectory of hyperactivity throughout development suggests a moderate stability (57). For instance, sleep problems operationalized as movements during the night (assessed by actigraphs) remain from childhood to adulthood (58). For many individuals, overt signs of hyperactivity decline from childhood into adulthood and may be confined with more subjective states such as mental restlessness, jitteriness, or impatience, indicating that the symptomatology undergoes substantial changes during the developmental course (1, 59, 60).

However, until to date, knowledge about the development of hyperactivity in the context of ADHD is limited and mainly based on retrospective self-reports of symptoms [e.g., Ref. (57)] which may well be affected by retrospective recall bias. To gain a more fine-grained understanding of the development of symptomatic hyperactivity and motoric activity across development, prospective longitudinal studies are needed that address symptom expressions as experienced by affected individuals themselves but also significant other people (e.g., parents, teachers, peers).

## Assessment Methods for the Detection of Symptomatic Hyperactivity and Motoric Activity in ADHD

Physical activity can be assessed with a variety of measures, including subjective self-reports via survey questionnaires or more frequent daily or hourly recalls, and more objective measures such as wearable sensors (i.e., pedometers, accelerometers including those built into mobile phones, heart rate sensors), doubly labeled water, and direct observation.

So far, there is no single gold standard of measuring activity across populations (e.g., children, adolescents, adults) and across different assessment purposes [e.g., activity status in populations, relationship of activity with short-term and long-term health and well being, clinical and intervention research (61)]. With widely varying time periods assessed (from minutes to days, weeks up to several years, over the lifespan) and activity definitions regarding activity dimension and intensity, findings obtained with different activity measures can be difficult to compare. One important consideration in choosing activity measures is assessment purpose: It is important to clearly specify the research purpose as well as the exact frequency, duration, and distribution of activity of interest and then choose the appropriate study design and assessment instrument. For example, studying sedentary behavior over several years to better understand the development of obesity will likely have to recur to questionnaires; whereas a study relating micro-movements of arms and legs to concurrent ADHD symptoms over a short time period would combine several sensors on arms and legs with frequent ADHD symptom assessments.

Self-report questionnaires are still the most commonly used method for measuring activity due to their cost effective application and low participant burden. Several reviews give an overview of available measures in children and adolescents (62–65) and adults (66–68). However, several other reviews also stated concerns about the validity of self-reported activity for youth and adults without clinical diagnoses as for instance ADHD (63, 67–70). Despite mostly acceptable reliability, the validity of PA questionnaires is still low to moderate. However, the reviews identified several questionnaires that showed both good reliability and acceptable validity [e.g., FPACQ, Flemish physical activity computerized questionnaire (71); PDPAR, previous day physical activity recall (72); RPAR, recess physical activity recall (73)]. To sum up, given that the validity of self-report questionnaires is limited in youth and adults without ADHD, their validity and reliability is even more questionable in children, adolescents, and adults with ADHD. This is because problems with inattention and impulsivity interfere with accurately noticing, memorizing, and reporting activity in a questionnaire, making reliable and valid self-reports even less likely.

Sensor-based activity assessment with pedometers and accelerometers has gained popularity in research (74–76) and in everyday life over the past years. As activity sensors shrink in size and have increasingly lasting batteries, they have become widely used in activity research in children, adolescents, and adults without ADHD. Wearable sensors require some buy-in from participants, as they have to be trained how to wear them – over the hip bone, usually putting them on in the morning and taking them off at night and during water-based activities – and have to give them back at the end of the study. Pedometers often do not store wear time in addition to steps and thus miss an important confounding variable for analysis; whereas accelerometers record wear time so that it can be included in analyses. Standard cut offs for valid days are at least 10 h of daily wear time (76). Among the disadvantages of pedometers are that they miss acceleration and speed of movement that should be especially interesting for understanding hyperactivity. Among their many advantages, pedometers are affordable (i.e., participants can even keep them after the study enabling continued self-monitoring), and they allow within-person comparisons. Pedometers and accelerometers are useful for measuring habitual activity in everyday life that is hard to capture in questionnaires because it evades conscious attention. Accelerometers can record more fine-grained activity information (i.e., speed, timing of movement) that may be particularly relevant for the assessment of hyperactivity. They can also detect movement of arms and legs if worn on wrist and ankle. However, there are also disadvantages of accelerometers and pedometers: Most devices miss water-based activities (i.e., swimming) and underestimate activities that do not involve movement of the part of the body where the sensor is located, as for example rowing or cycling with an accelerometer worn at the hip (77).

Smart phones have built-in accelerometers and can be used to measure activity. However, their assessment is less precise because the phone is not consistently worn in the same position (e.g., hand, pocket, bag). In the same vein, GPS assessments can be used but again are less precise about micro-movements. Heart rate sensors provide a comprehensive method of measuring physical exertion that captures many activities, not only vertical movement. Other methods for determining activity, such as doubly labeled water, multichannel devices combining accelerometers with respiration rate, electrocardiography, or electromyography and direct observation are expensive and can be burdensome for participants and are therefore more frequently used in smaller studies within the lab.

So far, there are few studies that have used sensor-based activity assessments in children and adolescents – and even fewer in those with ADHD. Thus, particularly in individuals with ADHD the cut-off points and algorithms for counting a movement as motion and classifying activity are still being developed. However, children diagnosed with ADHD differ from those without ADHD in the amount and intensity of their movements, as has been shown with different techniques of measuring movements, such as motion tracking systems using infrared motion analysis [(78); see also Ref. (79) for the Qb test], parent and teacher rating scales (80), and accelerometers (2). This knowledge has been used to identify children with ADHD with moderate accuracy measuring their activity with accelerometers over a 2-hour period (2) and with good accuracy measuring activity for 24 h (81).

In addition to comparisons of the amount and intensity of PA over a prolonged period of time, more fine-grained analyses of movements could also be informative. Modern accelerometers provide the opportunity to obtain data measured at very high resolutions (milli-G) and at very small time intervals (100th of a second). This allows not only for a measurement of the amount of activity, but also of more detailed qualitative differences in activities. These differences can already be detected with rather short measurement times. For example, it is possible to distinguish different kinds of activities by analyzing raw accelerometer data [e.g., Ref. (82, 83)]. In these studies, accelerometer data were accurately classified into different kinds of activities using Support Vector Machines [SVMs (84, 85)]. SVMs are machine learning techniques that allow for the classification of data into different categories. The great advantage of SVMs for this purpose lies in their ability to deal with highly complex and non-linear associations between accelerometer data and the corresponding categories of activity. Therefore, SVMs are perfectly suited to classify subjects as either having or not having ADHD. In recent years, this has already been done with quite some success with the use of different kinds of data, such as EEG (86, 87), inertial measurement units [IMU (88)], and MRI data (89–91).

These considerations indicate that fine-grained data from modern accelerometers, analyzed with SVMs, could be beneficial in two respects. First, they could be used to accurately identify children with ADHD with relatively little effort, in terms of both time and money. Second, those characteristics of accelerometer data that prove to be useful for distinguishing participants with and without ADHD could be further analyzed. By doing so, new insights about the nature of ADHD and hyperactivity could be obtained. These insights would go beyond the concept that was used in previous accelerometer studies on ADHD, namely that children with ADHD merely show higher amounts and intensities of activity. Hence, they could help to refine the concepts related to ADHD and hyperactivity (e.g., fidgeting, jitteriness), making them more objectively accessible, and less susceptible to subjective ratings.

## Modifying ADHD and ADHD Related Symptoms

Vigorous PA interventions in general address several areas that are problematic for children, adolescents, and adults with ADHD. For instance, short- and long-term interventions for increasing vigorous PA have led to improved mood and improved executive functioning (i.e., neuropsychological functions as for instance inhibition, shifting/task-switching, working memory), especially to improved inhibition performance in children, adolescents, and adults (92–94). Hence, enhancing vigorous PA could be an important additional treatment option for children with ADHD, ameliorating both comorbid affective disorders and deficits in executive functioning without potential negative side effects. Children diagnosed with ADHD might particularly benefit from PA interventions treating ADHD symptoms and comorbid problems due to various reasons: (a) PA might improve children’s emotional and social functioning in addition to having a positive effect on their cognitive functioning (95–97), (b) PA prevents health problems such as weight gain and obesity, which are common in children with ADHD due to impulsive behavior as for instance impulsive unhealthy snacking (98), (c) PA does not interact negatively with other therapy programs (e.g., medication with MPH, cognitive behavioral therapy), and (d) PA can easily be integrated into the everyday routine of children (e.g., in schools).

However, only few observational and single-case studies have reported improved attention and reduced hyperactivity (i.e., fidgetiness) in children with ADHD following regular PA sessions (99). Only recently, research investigated potential benefits of vigorous PA in children and adolescents with ADHD and found positive effects of various types of short- and long-term PA interventions on behavioral, (neuro-)cognitive, and comorbid symptoms associated with ADHD (99, 100). For instance, Medina et al. (101) examined the impact of running on a treadmill for 30 min in boys diagnosed with ADHD and showed improved sustained attention irrespective of medication use. More specifically, children improved on response time and vigilance in a Continuous Performance Test while decreasing in impulsivity after being physically active for 30 min. Tantillo et al. (102) tested the efficacy of treadmill walking versus quiet rest on the management of behavioral features of ADHD in 8- to 12-year-old children compared to matched comparison children. Improved motoric functions after exercise were found only in boys with ADHD. However, findings should be considered preliminary, as the sample size was rather small (i.e., 18 participants). Finally, Pontifex et al. (103) found that a single 20-min bout of exercise (i.e., again treadmill running) improved inhibitory performance and neurocognitive functions (i.e., EEG measures) in children with ADHD in particular.

Regarding long-term PA, Gapin and Etnier (99) found that higher levels of PA as measured by accelerometers were associated with better executive function performance in 18 boys with ADHD. Moderate-to-vigorous PA predicted the performance on the Tower of London planning task and was positively associated with other executive function measures. In a randomized study, Verret et al. (104) tested the effects of a moderate- to high-intensity PA program on fitness, cognitive functioning, and ADHD symptoms over 10 weeks in 21 children diagnosed with ADHD. Children in the treatment group showed better information processing and parents reported fewer attention problems as well as a lower total number of problems at follow-up than at baseline compared to children in the control group. In a pilot study, Smith et al. (105) investigated a daily 26-min continuous moderate-to-vigorous PA program before school that lasted for 8 weeks and found positive effects on inattention, hyperactive, and impulsive symptoms in children with ADHD: Response inhibition improved following the program, and ratings by parents, teachers, and program staff indicated overall improvements of motor, cognitive, and behavioral functioning in two thirds of participating children. Jensen and Kenny (106) randomly assigned 19 boys with ADHD stabilized on medication to a 20-session yoga group or a control group with cooperative activities. Both groups improved in hyperactive and impulsive behavior and the global DSM evaluation of ADHD. However, yoga decreased oppositional, restless, and impulsive behavior, and those in the yoga group who engaged in more home practice showed greater improvement for attention and affective lability.

The aforementioned studies demonstrate potential positive effects of PA interventions in children with ADHD. However, all studies are clearly underpowered and replication studies are warranted. Moreover, this claim for replication studies is underscored by a recent meta-analysis investigating the effects of acute bouts of PA in children with ADHD in laboratory and field studies, which revealed inconclusive results (107). Some laboratory studies found significant improvements on cognitive tasks (i.e., tasks measuring visual attention referring to symptomatic attention problems in ADHD and executive functions referring to underlying cognitive deficits in ADHD). One laboratory study (108) found a significant improvement in response times in a visual attention task, one study showed a maintenance of accuracy (109), and three studies showed a significant reduction of error rates but no influence on response times (103, 110, 111).

The meta-analysis also revealed that with respect to school settings, no effects of PA (i.e., as implemented in so called active lessons) on dependent variables such as measures of attention could be found. However, subgroups as elementary school children (112) seem to benefit from PA interventions. This is important because in this age group ADHD diagnoses are given frequently compared to other age groups. Furthermore, specific sports and activities as for instance coordinative exercises seem to be particularly helpful (113).

Thus, direct positive effects of acute and chronic PA interventions on ADHD symptomatic behavior including hyperactivity seem to be possible. Still, there is scarce research revealing heterogeneous results. An important research question that is still unanswered is whether there is a direct route of improving ADHD symptoms (i.e., hyperactivity) via PA or an indirect route improving for example executive function deficits leading to a subsequent improvement of ADHD symptoms. In the same vein it might also be the case that comorbid emotional deficits as for instance affective lability shown by children and adolescents with ADHD is altered via PA interventions leading to subsequent improvement of core ADHD symptoms (i.e., hyperactivity). More specifically, ADHD and affective problems are common co-morbidities in youths (114) and it might be the case that PA interventions target those affective problems and not the ADHD symptoms *per se*.

The association between vigorous PA and improved affect is well established and PA interventions have been shown to have positive effects on affect in healthy adults (115). While there is empirical evidence that children and adolescents accrue mental health benefits from PA interventions in general [e.g., Ref. (96)], until now, the specific link between physical activities and affect among children and adolescents has only been investigated in a few studies (116). A 10-year longitudinal study suggests that during adolescence, changes in leisure-time PA and negative affect are related inversely, that is, decreasing levels of PA are correlated with a rising prevalence of negative affect (117). In the same vein, a meta-analysis of studies investigating the depression-reducing effects of vigorous PA interventions on children and adolescents revealed effects in favor of the physically active group (118). Thus, vigorous PA is associated with lower levels of negative affect in children, adolescents, and adults in observational studies and randomized controlled trials.

Concretely, three studies have addressed the effects of PA on affective symptoms shown by children with ADHD so far. Jensen and Kenny (106) randomly assigned medicated boys with ADHD to a 20-session yoga condition or a control condition with cooperative leisure activities. Children in both conditions showed improvement in hyperactive and impulsive behaviors and in the global DSM evaluation of ADHD. Yoga decreased oppositional, restless, and impulsive behavior, and the children in the yoga condition who engaged in more home practicing of yoga showed greater improvement in attention and emotional stability. Kiluk et al. (119) found that participation in PA predicted less severe anxiety and depression in children with ADHD. Scores on parental reports of affect and behavior indicated that children with ADHD who participated in three or more sports displayed fewer symptoms of anxiety and depression compared to those children with ADHD who participated in fewer than three sports. Verret et al. (104) found in their exploratory but randomized study that teachers reported lower anxiety-depression scores and fewer social problems in children with ADHD after a 10-week PA program. In summary, PA interventions appear to improve not only executive functioning but also negative affect in children with and without ADHD.

## Summary

A bifactor model that represents a general ADHD symptom factor as well as independent specific symptom factors seems to be the best model to characterize the disorder. With regard to measuring hyperactivity as one of the ADHD symptoms only few studies have used sensor-based activity assessments in children and adolescents with ADHD. However, fine-grained accelerometer data analyzed with SVMs could potentially be useful to distinguish children with hyperactivity symptoms from those showing no hyperactivity symptoms. Moreover, further studies might also want to investigate the influence of medication (with MPH and Atomoxetine) and cognitive behavioral therapy on accelerometer data analyzed with SVMs. This is important because results could potentially inform about optimal treatments for individual children (i.e., tailored therapy). In order to gain further insight into the usefulness of PA interventions for children with motoric activity and hyperactivity as in children with diagnosed ADHD, it is important to investigate effects of PA regarding several aspects. First, the effects of everyday PA (i.e., biking to school, active lessons in school) and organized, structured sports need to be disentangled. Second, the dose–response relationship is in the need of being investigated: How often and for how long should children with ADHD take part in physical activities to receive an optimal level of their symptomatic behavior (i.e., fidgetiness in school). Third, interactions of medication with MPH or Atomoxetine and PA are understudied as well.

## Conflict of Interest Statement

The authors declare that the research was conducted in the absence of any commercial or financial relationships that could be construed as a potential conflict of interest.
